# A Structural Connectivity Disruption One Decade before the Typical Age for Dementia: A Study in Healthy Subjects with Family History of Alzheimer’s Disease

**DOI:** 10.1093/texcom/tgab051

**Published:** 2021-08-27

**Authors:** F Ramírez-Toraño, Kausar Abbas, Ricardo Bruña, Silvia Marcos de Pedro, Natividad Gómez-Ruiz, Ana Barabash, Ernesto Pereda, Alberto Marcos, Ramón López-Higes, Fernando Maestu, Joaquín Goñi

**Affiliations:** Laboratory of Cognitive and Computational Neuroscience, Center for Biomedical Technology, Universidad Politécnica de Madrid, Madrid 28223, Comunidad de Madrid, Spain; Department of Experimental Psychology, Universidad Complutense de Madrid, Madrid 28223, Comunidad de Madrid, Spain; Purdue Institute for Integrative Neuroscience, Purdue University, West-Lafayette, IN 46202, USA; School of Industrial Engineering, Purdue University, West Lafayette, IN 46202, USA; Laboratory of Cognitive and Computational Neuroscience, Center for Biomedical Technology, Universidad Politécnica de Madrid, Madrid 28223, Comunidad de Madrid, Spain; Department of Experimental Psychology, Universidad Complutense de Madrid, Madrid 28223, Comunidad de Madrid, Spain; Networking Research Center on Bioengineering, Biomaterials, and Nanomedicine (CIBER-BBN), Madrid 28029, Comunidad de Madrid, Spain; Laboratory of Cognitive and Computational Neuroscience, Center for Biomedical Technology, Universidad Politécnica de Madrid, Madrid 28223, Comunidad de Madrid, Spain; Facultad de Educación y Salud, Universidad Camilo José Cela, Madrid 28010, Comunidad de Madrid, Spain; Sección Neurorradiología, Servicio de Diagnóstico por Imagen, Hospital Clínico San Carlos, Madrid 28040, Comunidad de Madrid, Spain; Endocrinology and Nutrition Department, Hospital Clinico San Carlos and Instituto de Investigación Sanitaria del Hospital Clínico San Carlos, Madrid 28040, Comunidad de Madrid, Spain; Centro de Investigación Biomédica en Red de Diabetes y Enfermedades Metabólicas Asociadas, Madrid 28029, Comunidad de Madrid, Spain; Laboratory of Cognitive and Computational Neuroscience, Center for Biomedical Technology, Universidad Politécnica de Madrid, Madrid 28223, Comunidad de Madrid, Spain; Electrical Engineering and Bioengineering Group, Department of Industrial Engineering & IUNE & ITB, Universidad de La Laguna, Santa Cruz de Tenerife 38205, Spain; Neurology Department, Hospital Clinico San Carlos and Instituto de Investigación Sanitaria del Hospital Clínico San Carlos, Madrid 28040, Comunidad de Madrid, Spain; Department of Experimental Psychology, Universidad Complutense de Madrid, Madrid 28223, Comunidad de Madrid, Spain; Laboratory of Cognitive and Computational Neuroscience, Center for Biomedical Technology, Universidad Politécnica de Madrid, Madrid 28223, Comunidad de Madrid, Spain; Department of Experimental Psychology, Universidad Complutense de Madrid, Madrid 28223, Comunidad de Madrid, Spain; Networking Research Center on Bioengineering, Biomaterials, and Nanomedicine (CIBER-BBN), Madrid 28029, Comunidad de Madrid, Spain; Purdue Institute for Integrative Neuroscience, Purdue University, West-Lafayette, IN 46202, USA; School of Industrial Engineering, Purdue University, West Lafayette, IN 46202, USA; Weldon School of Biomedical Engineering, Purdue University, West Lafayette, IN 46202, USA

**Keywords:** Alzheimer’s disease, cascading network failure, diffusion-weighted imaging, early detection, family history of Alzheimer’s disease

## Abstract

The concept of the brain has shifted to a complex system where different subnetworks support the human cognitive functions. Neurodegenerative diseases would affect the interactions among these subnetworks and, the evolution of impairment and the subnetworks involved would be unique for each neurodegenerative disease. In this study, we seek for structural connectivity traits associated with the family history of Alzheimer’s disease, that is, early signs of subnetworks impairment due to Alzheimer’s disease.

The sample in this study consisted of 123 first-degree Alzheimer’s disease relatives and 61 nonrelatives. For each subject, structural connectomes were obtained using classical diffusion tensor imaging measures and different resolutions of cortical parcellation. For the whole sample, independent structural-connectome-traits were obtained under the framework of *connICA*. Finally, we tested the association of the structural-connectome-traits with different factors of relevance for Alzheimer’s disease by means of a multiple linear regression.

The analysis revealed a structural-connectome-trait obtained from fractional anisotropy associated with the family history of Alzheimer’s disease. The structural-connectome-trait presents a reduced fractional anisotropy pattern in first-degree relatives in the tracts connecting posterior areas and temporal areas.

The family history of Alzheimer’s disease structural-connectome-trait presents a posterior–posterior and posterior–temporal pattern, supplying new evidences to the cascading network failure model.

## Introduction

It is well established that Alzheimer’s Disease (AD) can be considered a long-duration neuropathological process starting about 20 years before the appearance of the typical clinical symptoms as it is episodic memory impairment. It would be ideal to identify a biomarker indicating the relative risk for the development of dementia, as early in time as possible. For this reason, there is an increased interest in the study of the preclinical stages of the AD continuum, specifically in populations at risk with no symptoms, neither brain pathology. A typical example of this population at risk, are the relatives of AD patients. It has been found that first-degree relatives of AD who are carriers of ɛ3/ɛ4 showed a lifetime risk of 46.1% and up to 61.4% in ɛ4/ɛ4 carriers. However, this risk decreases to 29.2% in first-degree relatives carrying ɛ3/ɛ3 alleles (Martinez et al. 1998; Bendlin et al. 2010a). Therefore, this population is of great interest for identifying early neurophysiological characteristics of the disease that could open new vistas for intervention (Ramírez-Toraño et al. 2020). To accomplish this challenging aim it is necessary to approach this complex disease from new perspectives.

In the recent years, the concept of the brain organization has shifted to a complex system where different subnetworks support the human cognitive functions. In this framework, neurodegenerative diseases would not solely affect the brain at a locally molecular level but the normal functions and interactions among these subnetworks. The evolution of impairment and the subnetworks involved would be unique for each neurodegenerative disease (Seeley et al. 2009). In the case of sporadic AD, the network-level malfunction would start in highly connected posterior regions progressing to hyperconnectivity with ventral and anterior areas (Jones et al. 2016). This model, denominated cascading network failure, accords with the AD pathophysiology observed with other biomarkers.

Diffusion-weighted imaging (DWI) is a specific technique of magnetic resonance imaging (MRI) that measures the movement of water particles in the brain (Le Bihan and Breton 1985). The DWI information and the properties of water movement in the different tissues of the brain allow extracting more complex information about the structural integrity of the brain. Diffusion tensor imaging (DTI) technique characterizes the magnitude, the degree of anisotropy, and the orientation of water diffusion and thus, test the structural integrity of the brain. DTI technique has been widely used in the study of AD. The most reported observation in advanced AD stages and mild cognitive impairment (MCI) is a decreased in fractional anisotropy (FA) and an increase in mean diffusivity (MDiff), especially in the hippocampal cingulum and in the posterior, temporal and parietal areas white matter (WM) (Bozzali et al. 2002; Douaud et al. 2011; Nir et al. 2013; Mayo et al. 2019). Fewer studies might be found with cognitively healthy population at risk of developing AD but still, the reports show an initial impairment in posterior and temporal areas (Gold et al. 2010; Smith et al. 2010; Bendlin et al. 2010b).

Nevertheless, since no considerable impairments are expected in cognitively healthy population at risk, the classical straightforward comparison techniques might obscure subtle patterns of connectivity. To address this matter, Amico et al. (2017) developed an independent component (IC) approach that untangle different patterns of brain connectivity present in the population under study. This approach estimates independent connectivity patterns (or traits) present in the whole population without any stratification of subjects or supervised classification into groups. Instead, the presence of traits on each subject is a posteriori evaluated in order to assess possible associations with cognition or behavior. This framework has been used to identify connectivity traits related to levels of consciousness (Amico et al. 2017), to mild cognitive impairment and AD (Contreras et al. 2017), structural-functional connectivity traits that support cognitive tasks (Amico and Goñi 2018) and, more recently, family history of alcoholism (Amico et al. 2020).

In this study, we estimate structural ICs under the framework of *connICA* using classical DTI measures and different resolutions of cortical parcellation. We then test the association of the obtained traits with a collection of demographic, genetic, neuropsychological, and neurophysiological factors that have been proven to be related to AD and structural connectivity. Specially, we seek for structural connectivity traits associated with the family history of AD.

## Material and Methods

### Participants

Two hundred and sixty two healthy older adults were recruited from local hospitals, via advertisements in the Fulbright alumni association, in the “Asociación Española de Ingenieros de Telecomunicación Delegación de Madrid”, as well as in public media. Exclusion criteria for the current study comprised: (1) history of psychiatric or neurological disorders or drug consumption in the last week that could affect MEG activity; (2) evidence of infection, infarction or focal lesions in a T2-weighted MRI scan; (3) alcoholism or chronic use of anxiolytics, neuroleptics, narcotics, anticonvulsants, or sedative hypnotics; (4) Mini-Mental State Examination (MMSE) score below 27; (5) subjective cognitive complaints; (6) unusable T1-weighted image or DWI. All participants underwent a comprehensive battery of neuropsychological tests, a blood extraction procedure, and an MRI scan. None of the participants included in this study meets the diagnosis criteria for AD, MCI, or preclinical stages of AD (Albert et al. 2011; Gosche et al. 2002; McKhann et al., 2011; Sperling et al. 2011). The demographic, neuropsychological, and neurophysiological data of each subject is included in Supplementary Table 2. When specifically looking at the temporal cortical thickness (average thickness across entorhinal, fusiform, inferior temporal, and middle temporal), 3 subjects out of 184 had a value below the standard interquantile range (2.61–2.80) reported by Jack et al. (2017). However, none of the 3 subjects fulfilled the criteria for AD, MCI, or preclinical stages of AD.

All participants signed an informed consent. The “Hospital Clínico San Carlos” Ethics Committee approved this study, and the procedure was performed in accordance with international approved guidelines and regulations.

The final sample in this study consisted of 184 participants: 123 first-degree AD relatives and 61 nonrelatives. First-degree relatives were defined as being direct descendants or siblings of a patient with AD. Relatives of AD patients were required to provide a medical report indicating the diagnosis of the patient following the NINCDS-ADRDA criteria (McKhann et al. 1984). The characteristics of the sample are displayed in Table 1.

**Table 1:** Demographic characteristics of the population

	AD relatives	Controls	Bootstrap *P* value	Bootstrap effect size
N	123	61		
Age	57.88 ± 6.85	62.72 ± 9.31	(0.0524–1.0000)	(0.0000–0.3548)
*APOE* ε4 carriers	46 (+)/77 (−)	13 (+)/48 (−)	(0.0716–1.0000)	(0.0000–0.1631)
Sex	42 M/81 F	25 M/36 F	(0.0538–1.0000)	(0.0000–0.1746)
Years of education	15.5040 ± 3.8376	16.9180 ± 3.8508	(0.0529–0.9806)	(0.0044–0.3540)
MoCA	26.1157 ± 2.7792	25.9993 ± 2.5375	(0.0515–0.9960)	(0.0009–0.3561)
TPA	1.4411 ± 1.1921	1.5465 ± 1.1567	(0.0538–0.9722)	(0.0063–0.3527)
Average cortical thickness	2.3704 ± 0.0724	2.3517 ± 0.0780	(0.0558–1.0000)	(0.0000–0.3496)
Hippocampi volume	3820.99 ± 377.23	3751.36 ± 445.17	(0.0509–0.9997)	(0.0001–0.3570)

Values are presented as mean ± standard deviation. TPA values are normalized by actigraphy wear time. The cortical thickness is expressed in *millimeters*. The hippocampi volume is the average of left and right hippocampi volumes. The 2 last columns present the minimum and the maximum *P* value and effect size of each predictor across the 100 samplings. (+) *APOE* ε4 carriers; (−) non *APOE* ε4 carriers; M = male; F = female; MoCA = Montreal Cognitive Assessment; TPA = total physical activity.

### APOE Genotype Test

DNA was extracted from whole-blood samples of the participants of this study. As previously described in (Cuesta et al. 2015), APOE haplotype was determined by analyzing SNPs rs7412 and rs429358 genotypes with TaqMan assays using an Applied Biosystems 7500 Fast Real Time PCR machine (Applied Biosystems, Foster City, CA, USA). A genotyping call rate over 90% per plate, sample controls for each genotype and negative sample controls were included in each assay. Three well-differentiated genotyping clusters for each SNP were required to validate results. Intra- and interplate duplicates of several DNA samples were included.

### MRI Data

#### Image Acquisition

Each subject T1-weighted MRI image was acquired in a General Electric 1.5 Tesla system. A high-resolution antenna was employed together with a homogenization Phased array Uniformity Enhancement filter (Fast Spoiled Gradient Echo sequence, TR/TE/TI = 11.2/4.2/450 ms; flip angle 12°; 1 mm slice thickness, 256 × 256 matrix and field of view (FOV) 25 cm).

The acquisition parameters for DWI were as follows: TE/TR 96.1/12000 ms; NEX 3 for increasing the SNR; 2.4 mm slice thickness, 128 × 128 matrix, and 30.7 cm FOV yielding an isotropic voxel of 2.4 mm; 1 image with no diffusion sensitization (i.e., T2-weighted b0 images); and 25 DWI (b = 900 s/mm^2^). Data were recorded with a single shot echo planar imaging sequence.

#### T1 Processing

Each subject T1-weighted MRI image was processed using FreeSurfer 6.0 *recon-all* procedure as described in (Dale et al. 1999; Fischl et al. 1999a, 1999b, 2001; Ségonne et al. 2004, 2007). First, this procedure performs a motion correction, corrects for intensity nonuniformity and performs and intensity normalization. Then, it performs a segmentation of the different brain tissues and it constructs a cortical surface mesh for each T1. It registers an inflated sphere version of this cortical mesh to a common surface-space. Finally, it uses an anatomical atlas (this atlas must be also an inflated version of the surface atlas and register to the common surface-space) to assign a neuroanatomical label to each native brain voxel. In this study, we have used the cortex parcellation scheme proposed by (Schaefer et al. 2018). This parcellation scheme divides the cortex into 7 functional networks with 10 different levels of spatial granularity (from 100 parcels up to 1000 parcels). For completeness of those atlases, subcortical regions were added as obtained using the *FIRST* command provided by FSL software (Patenaude 2007). An example of a whole brain structural connectome organized into hemispheres and resting-state networks (RSN) is shown in Supplementary Figure 1.

As last step, we obtained the cortical thickness using the FreeSurfer software and we registered the T1-space cortical atlas to each subject’s DWI-space using the linear registration tool (“flirt” command with 7 degrees of freedom) as provided by FSL software (Jenkinson and Smith 2001; Jenkinson et al. 2002).

#### DWI Processing

The DWI data were processed using the MRtrix3 software (Tournier et al. 2019). The DWI processing was compounded from the following sequential steps: (1) DWI denoising (Veraart et al. 2016), Gibbs-ringing artifacts removal (Kellner et al. 2015), eddy current and movements correction (Andersson and Sotiropoulos 2016), DWI bias field correction; (2) generation of a tissue-type segmented image appropriate for anatomically constrained tractography (Smith et al. 2012); (3) estimation of WM, gray matter (GM) and cerebrospinal fluid (CSF) response functions for each subject (Dhollander et al. 2016). The final response function used for the whole sample is the average of all subjects’ response functions; (4) Single-Shell 3-Tissue CSD (SS3T-CSD) was performed to obtain WM-like FODs as well as GM-like and CSF-like compartments in all voxels (Dhollander and Connelly 2016), using MRtrix3Tissue (https://3Tissue.github.io); (5) Multitissue informed log-domain intensity normalization (Raffelt et al. 2017); (6) generation of the tractogram (25 millions streamlines, maximum tract length = 250, FA cutoff = 0.06, dynamical seeding) (Tournier and Calamante 2010); SIFT2 tractography optimization (Smith et al. 2015).

For each tractography, we obtained a set of structural connectomes corresponding to each of the 10 Schaefer parcellations. In particular, for each parcellation, structural connectomes were estimated based on the 5 following measures: number of streamlines (NoS), FA, axial diffusivity (ADiff), MDiff, and radial diffusivity (RDiff). Overall, this process results in 50 structural connectomes (SC) for each subject (5 structural measures and 10 parcellations).

### Independent Component Analysis of Structural Connectomes

**Figure 1:**
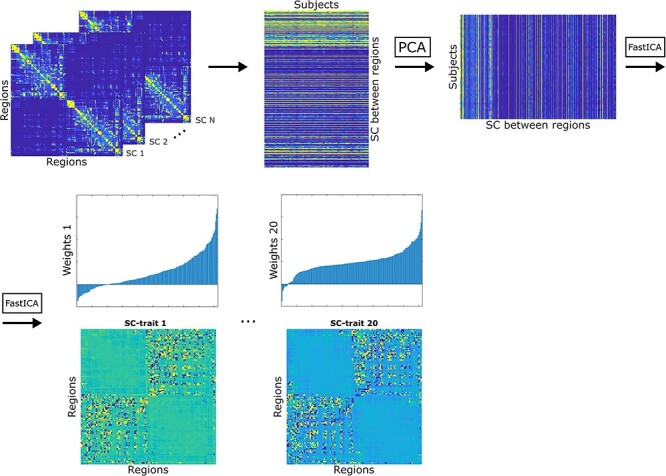
Scheme of the *connICA* framework applied to structural connectomes. The original structural connectomes are vectorized and assembled to create a structural connectivity matrix for the whole population. The matrix is preprocessed by means PCA and an ICA is performed. The output consist of 20 SC-traits, presents in the whole population, and their associated weights, quantifying the presence of each SC-trait in each subject. SC = structural connectome; PCA = principal component analysis; ICA = independent component analysis.

The workflow to obtain ICs from structural connectomes is based on the *connICA* methodology used for functional connectomes (Amico et al. 2017). For a single parcellation and structural measure, each SC was transformed into a column vector keeping only the upper triangular part of the SC. The SC vectors of the whole population were concatenated into a single matrix. To avoid possible sources of noise, we performed a principal component analysis (PCA) and reconstructed the whole-population matrix using the number of components needed to explain the 95% of the variance. Note that the number of PCA components needed is different for each measure and parcellation. Over this reconstructed matrix, we performed an independent component analysis (ICA) by running the *FastICA* algorithm (Hyvärinen 1999). The number of ICs was set to 20. For each IC, we obtained 2 output vectors: the first output vector will be referred to as SC-trait and it represents the IC itself; the second output vector will be referred to as weights and it quantifies the importance or presence of this SC-trait in each subject. A scheme of this framework is shown in Figure 1. This process was repeated for all combinations of structural measures and parcellations schemes.

The nondeterministic nature of the *FastICA* algorithm (Hyvärinen 1999) represents a problem for the consistency of the solutions obtained. In order to get consistent solutions, we ran the *FastICA* algorithm 500 times and we imposed 2 constraints to keep only the “robust” SC-traits: first, it has to be present, at least, in 75% of the runs; second, a SC-trait from one run “is present” in another run if it has a correlation of 0.7 or higher with any of the SC-traits of that second run.

### Statistical Analysis

The first statistical analysis compares the presence of each SC-trait in the relatives and nonrelatives groups by means of a *t*-test. A *t*-test was performed using the weights of each of the robust SC-traits obtained. To address the multiple comparison problem, a false discovery rate (FDR) correction was applied taking into account the number of robust SC-traits found, the number of structural measures, and the parcellation resolution. The significance level for the FDR correction was set to *q* < 0.05.

The second statistical analysis consists in an incremental multiple lineal regression (MLR) model using as response the weights of the robust SC-traits aforementioned and 9 predictors. The selected predictors are a collection of demographic, genetic, neuropsychological, and neurophysiological factors that have been proven to be related to AD and SC. The last predictor added is family history of AD so that we can evaluate the isolated contribution of familial history when accounting for all other 8 predictors. Predictors are presented in Table 1.

To avoid possible biases driven by the unbalanced stratification of our cohort into family history (*N* = 123) and controls (*N* = 61), the MLR model was estimated using a sampling without replacement scheme. For each bootstrap iteration, 61 first-degree relatives are selected randomly without replacement. Only iterations where the 61 first-degree relatives selected show not significant differences with the control group in any of the remaining 8 predictors are kept for further evaluation. With this constraint, we avoid any group bias on the predictors that might interact or confound with being first-degree relatives or not. This procedure is repeated until obtaining 100 unbiased samplings (runs) of the family history group. For each sampling, the statistics of the corresponding MLR model are saved. Results are summarized by averaging the statistics across runs, namely: predicted values, standard residuals, predictability of the model (${R}^2$), regression coefficients of each predictor (β), and *t*-statistic of each predictor. The *P* value associated to each predictor is calculated using the averaged *t*-statistic. To address the multiple comparison problem, a FDR correction was applied taking into account the number of robust SC-traits found, the number of predictors, the number of structural measures, and the parcellation resolution. The significance level for the FDR correction was set to *q* < 0.05.

## Results

In this section, we present the structural connectivity patterns found in a young cognitively healthy population using the *connICA* technique. This technique reveals ICs of structural connectivity, SC-traits, present in the whole population under study without any prior stratification. First, we study the presence of the robust SC-traits in the relatives and nonrelatives groups. Finally, we study the association of these SC-traits with demographic, neuropsychological, and neurophysiological variables of interest by means of a multiple linear regression.

### Robust SC-Traits

A SC-trait is defined as robust when it is present, at least, in 75% of the 500 runs as defined in (Amico et al. 2017). We explored the presence of robust SC-traits across structural measures and Schaefer parcellations. Results are shown in Table 2. Note that for the cases where no robust SC-traits were found, no further investigation on the traits or their possible association with demographic and/or cognition was performed.

### Presence of the SC-Traits

**Figure 2:**
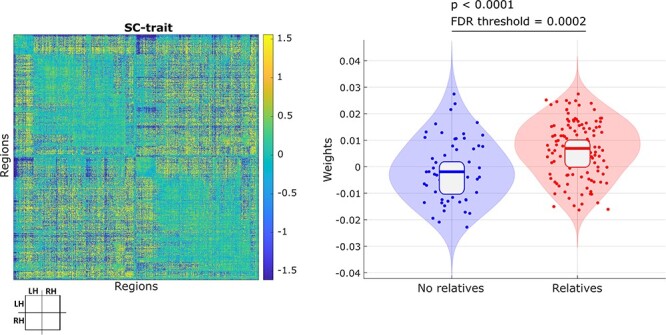
Family history of AD SC-trait. On the left, the matrix represents the connectivity patterns of connectivity of the SC-trait. The positive values (yellow) represent an increased FA connectivity in the AD relatives group and the negative values (blue) represent a decreased FA connectivity in the AD relatives group. On the right, the weights of this SC-trait for each subject, that is, the presence of this SC-trait in the connectivity pattern of each subject. The violin plots present the weights distribution of each group with the quartiles and the median. The *P* value is the result of a two-tailed *t*-test. LH = left hemisphere; RH = right hemisphere; FA = fractional anisotropy.

One SC-trait survived the FDR correction when compared the weights of the relatives and nonrelatives groups. The SC-trait associated with family history of AD is obtained with the FA measure and the 800 areas parcellation. This SC-trait shows a pattern of altered interhemispheric connectivity, with an important negative cluster in the interhemispheric temporal-occipital connections and a more widespread positive alteration. The presence of the SC-trait (i.e., the weights obtained by *connICA*) in the whole population is 0.0039 ± 0.0116 (mean ± std), whereas in the first-degree relatives group is 0.0065 ± 0.0108 and in the control group is −0.0012 ± 0.0115. Figure 2 shows the significant SC-trait in its matrix form and the presence of the SC-trait in each subject.

**Table 2:** Number of robust SC-traits found for each structural measure and atlas resolution

Structural measures	Atlas resolution									
	100	200	300	400	500	600	700	800	900	1000
NoS	3	9	8	5	8	8	7	6	2	8
FA	0	5	11	7	6	9	10	10	10	10
ADiff	0	15	12	11	11	12	10	9	7	9
MDiff	–	–	–	–	–	–	–	–	–	–
RDiff	–	–	–	–	–	–	–	–	–	–

The number of robust SC-traits is presented. The dash symbol (‘-’) indicates the cases where was not possible to obtain 20 independent components. NoS = number of streamlines; FA = fractional anisotropy; ADiff = axial diffusivity; MDiff = mean diffusivity; RDiff = radial diffusivity.

### Multiple Linear Regression

The SC-trait associated with family history of AD is further analyzed. The SC-trait was considered for associations with different characteristics of the subjects. To do so, we used demographic, genetic, neuropsychological, and neurophysiological factors as predictors in a multilinear regression model to predict the subjects’ weights of the SC-trait.

#### Sampling without Replacement Scheme

As imposed by the sampling scheme, the 2 groups did not differ in any of the predictors across the 100 samplings. The minimum and maximum *P* value and effect size for each predictor across the 100 samplings are presented in Table 1.

#### Multiple Linear Regression Results

**Figure 3:**
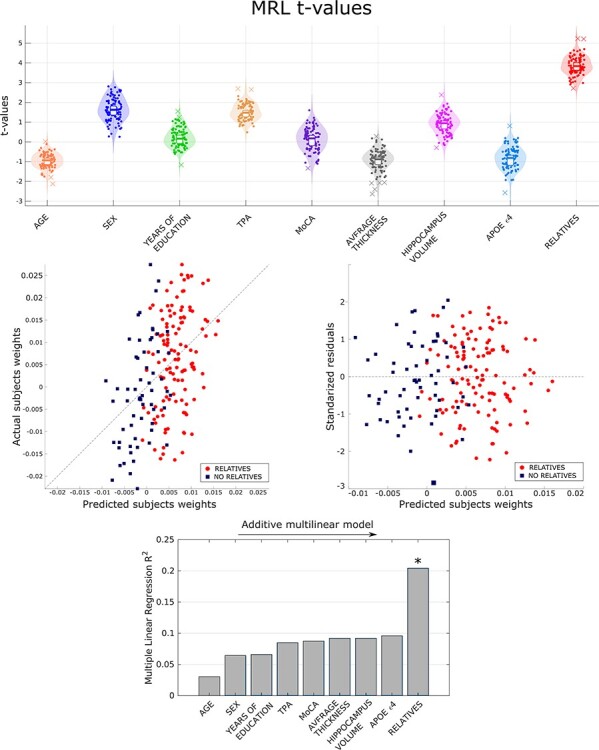
Multiple linear regression model. On the top panel, the *t*-statistic associated to each of the 9 predictors used to define the model. On the mid panel, the quality of the model, that is, the real weight of each subject versus the weight predicted by the model and the error associated to each prediction. On the bottom panel, the contribution of each predictor to the overall predictability of the model.

The predictability of this MLR model using the 9 aforementioned predictors was ${R}^2=0.2121$. We assessed the relative contribution of each predictor to the general predictability of the model adding sequentially the predictors to the MLR model. The most relevant predictors for the MLR model were family history of AD (relative contribution of 0.1084), sex (0.0341), and age (0.0303). The only predictor significantly associated with this SC-trait is family history of AD ($t(112)=3.8713$, $p=0.0002$) with a standardized regression coefficient $\beta =0.3423\pm 0.0372$ and an explained variance of ${R}^2=0.1084$. The positive sign of the regression coefficient indicates that first-degree relatives have a greater presence of this SC-trait. The value of the FH ${R}^2$ indicates a strong variation in the SC-trait weights due to the risk factor of positive family history of AD. The information of the MLR model is visually summarized in Figure 3. To see the complete information of the MLR model refer to Supplementary Table 1.

#### Anatomical Areas of the Family History of AD SC-Trait

In order to comprehend the implications of the SC-trait, we condensed the SC-trait into the 7 functional networks defined by (Schaefer et al. 2018). First, the SC-trait is split into 2 subtraits, one reflecting enhanced integrity and the other reflecting diminished integrity. As shown in Figures 2 and 3, the presence of this SC-trait is significantly higher in first-degree relatives. Hence, the enhanced integrity represents a pattern of increased integrity in the first-degree relatives group and the diminished integrity represents a pattern of decreased integrity in the first-degree relatives group. Hence, from now on, we will refer to those subtraits as FH enhanced integrity and FH diminished integrity respectively. For each subtrait, we retained the giant component of the connectivity pattern, hence, discarding spurious edges involving one or a few brain areas (Goñi et al. 2013). Then, we estimated the presence of each RSN as the sum of the weights of the within and between RSN edges divided by their respective total number of edges. The denominator takes into account the RSNs size so that results are comparable within and between RSNs. Our hypothesis includes that FH subjects will not show an integrity gain in connectivity respect to the control group. Instead, we expect them to possibly have a loss of structural integrity (Gold et al. 2010; Smith et al. 2010; Bendlin et al. 2010b). In accordance with this hypothesis, we used the highest value of RSNs presence in FH enhanced subtrait (0.6076) to build our null model thresholds of spurious FA values at the networks level. In the same manner, we used the highest value of RSNs presence in FH diminished subtrait (1.4720) to validate our hypothesis (i.e., any value of the FH enhanced subtrait is greater than this value).

**Figure 4:**
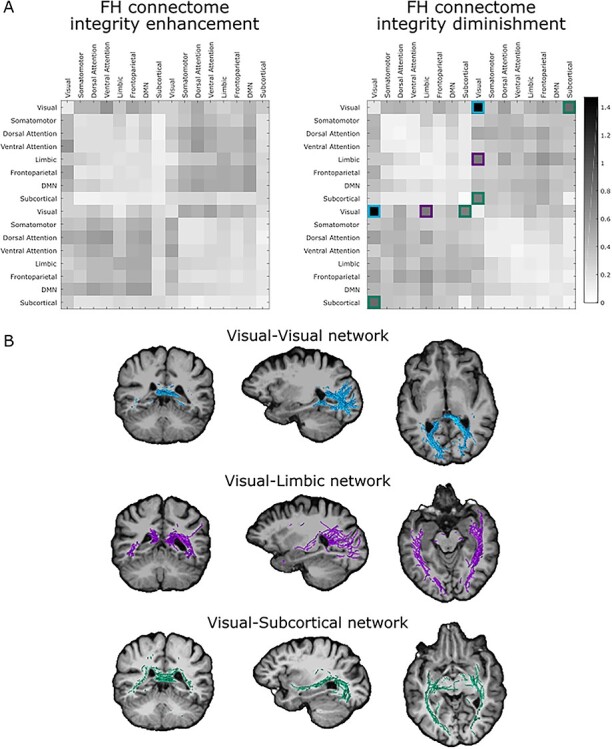
Anatomical visualization of the family history of AD SC-trait. The family history of AD SC-trait is split in 1 positive and 1 negative matrix, excluding spurious connections. The most active networks are estimated as the sum of the connectivity patterns of the areas belonging to that network divided by the numbers of areas. The most active networks were found in the negative matrix and they were the visual–visual network (blue), the visual-limbic network (purple), and the visual-subcortical network (green). The tracts of these networks are visually presented over an MRI for clarity purposes. DMN = default mode network.

All values of the FH enhanced subtrait are lower than the threshold applied (1.4720). The 3 over-represented within and between RSNs in the FH diminished subtrait are: visual–visual, visual–limbic and visual–subcortical. Results are shown Figure 4A. In order to get an anatomical visualization of the results, we extracted the streamlines from the original tractography for each over-represented result (Fig. 4B).

This process revealed that the most active functional networks were found in the negative matrix, that is, areas where the first-degree relatives present a reduced connectivity pattern, involving the visual–visual network, the visual limbic network and the visual–subcortical network as defined by (Schaefer et al. 2018). Anatomically, we observed a decreased pattern of connectivity between posterior regions, between posterior and superior temporal regions (including the insula), and between posterior regions and the amygdala.

## Discussion

In this work, the presence of disrupted structural connectivity patterns associated with the family history of AD has been addressed. The population under study is about one decade younger compared with the average onset of the sporadic AD and present no differences in relevant markers of AD, namely: *APOE* ε4 carriage, demographic characteristics, neuropsychological performance, hippocampi volume, and cortical thickness. The SC-traits were obtained for 5 different DTI measures and 10 different resolutions of the brain parcellation using the *connICA* technique. The SC-traits obtained with this method are present in the whole population without any stratification of the subjects and without knowing if they have any relation with the variables of interest. The posterior MLR analysis revealed a SC-trait obtained from FA associated with the family history of AD. The SC-trait presents a reduced FA pattern in first-degree relatives in the tracts connecting posterior areas and temporal areas.

The essential of this study is the exploratory analysis of structural connectivity in young healthy population to reveal early effects of sporadic AD. With this purpose, we have selected the most used DTI measures in the literature and a brain parcellation scheme that allows us to obtain different levels of resolution of the same defined brain functional networks (Schaefer et al. 2018). Furthermore, the expected changes in the first-degree relatives population, if any, might be so subtle that straightforward connectivity matrix comparisons may loss early AD signs in young population at risk. *connICA* technique extracts independent structural connectivity patterns (SC-traits) present in the population without any kind of stratification or a priori assumption about the population. Later, the association of the SC-traits to different variables of interest is further analyzed. This framework has been previously used in different experiments such as, levels of consciousness (Amico et al. 2017), mild cognitive impairment and AD (Contreras et al. 2017), structural-functional connectivity traits that support cognitive tasks (Amico and Goñi 2018) and family history of alcoholism (Amico et al. 2020). Table 2 shows the diverse number of SC-traits obtained for each parcellation resolution and DTI measure, indicating the benefits of our framework for an exploratory study.

The MLR models the relationship between the SC-traits and the set of demographic, genetic, neuropsychological, and neurophysiological factors relevant for sporadic AD. Among all the SC-traits, one showed a significant association to the family history of AD. Furthermore, family history of AD is the only significant predictor for this SC-trait, addressing current discussions in AD literature. The fact that the 2 groups do not differ in hippocampi volume or cortical thickness and the fact that these 2 predictors are not associated with this SC-trait, may support the idea that WM alterations are not caused by Wallerian degeneration secondary to GM atrophy (Gold et al. 2010; Zhuang et al. 2012; Caballero et al. 2018). Neither being an *APOE* ε4 carrier is associated with this SC-trait, a surprising outcome previously reported in the literature (Chalmers et al. 2005; Bendlin et al. 2010b). This same outcome was found in this population in a previous study of functional connectivity using magnetoencephalography (Ramírez-Toraño et al. 2020). The null contribution of *APOE* ε4 to this SC-trait does not imply that *APOE* ε4 has no effect on the progression of AD. It only can be inferred the presence of an abnormal connectivity pattern related to the risk factor of family history of AD. APOE ε4 has been associated with the deposition of amyloid plaques, which could be a different pathway of AD pathology than the disrupted WM integrity. These 2 pathways could be interacting at some point in the pathology but seems to be relatively independent at this stage of the AD continuum.

The family history of AD SC-trait presented a decreased connectivity pattern between posterior areas, between posterior areas and temporal areas, and between posterior areas and the amygdala. Decreased FA has been reported in preclinical AD, amnestic MCI and clinically diagnosed AD dementia patients (Chua et al. 2008, 2009; Wang et al. 2009; Bendlin et al. 2010b; Liu et al. 2011; Kantarci et al. 2017). Furthermore, the visual–limbic SC-trait presented in this study reminds of the ventral cortical pathway defined by Mishkin et al. (1983), which is related with high-order visual recognition. Damage in this cortical pathway could lead to difficulties in face-recognition tasks, difficulties that worsen with the AD progression (Huang et al. 2020). These results might demonstrate an early structural impairment before the presence of any clinical or neurophysiological alteration. The association of this finding with the typical pathophysiology of the disease is hard to establish with the current data. Nevertheless, the early disruption of the WM integrity could be due to an initial effect of the tau-pathology associated with AD since pyramidal neurons in the temporal cortex prone to be particularly vulnerable to tau-pathology (Hof et al. 1990). The hyper phosphorylation of the tau-protein affects the structure of the axonal microtubules and consequently the axon structure. This effect could be lastly seen in the reduction of the tracts integrity by DTI. In fact, this relationship has been already demonstrated in AD patients with posterior cortical atrophy (Sintini et al. 2019). This hypothesis needs to be tested by mixing SC and tau-PET scans in subjects with family history of AD. As a final remark, we observed widespread effects of WM integrity enhancement and diminishment (Fig. 2). Although the enhancement effect associated to FH could be due to actual neurophysiological changes, it is more probable that this effect is caused by the uncertainty introduced by the fitted model of local diffusion and by the intrinsic uncertainty associated with the estimation of DTI parameters (Behrens et al. 2003; Jones 2003; Polders et al. 2011). The methodology employed in this study, that is, the use of the highest value in the FH enhanced subtrait, considers this effect as a background noise due to intrinsic uncertainty associated with the DTI parameters, and set the threshold for significant diminishment changes associated with FH (Fig. 4).

Recently, (Jones et al. 2016) proposed a model for AD progression defined as a cascading network failure. According to this model: “The failure begins in the posterior default mode network, which then shifts processing burden to other systems containing prominent connectivity hubs.”. The “systems containing prominent connectivity hubs” would be the temporal and frontal areas. The family history of AD SC-trait presented in this study might be a supporting evidence of this model. The family history of AD SC-trait resembles this model definition, presenting posterior–posterior and posterior–temporal abnormal connectivity patterns in the population at increased risk of AD.

To conclude, this study has presented the family history of AD SC-trait, an abnormal FA connectivity pattern related to the family history of AD. This SC-trait cannot be explained by any of the other relevant factors of AD such as *APOE* ε4 carriage, demographic characteristics, neuropsychological performance, and neurophysiological characteristics. This SC-trait presents a posterior–posterior and posterior–temporal pattern, supplying new evidence to the cascading network failure model.

There are 3 main lines of future work for this study. First, interesting results have been found in the same population regarding functional connectivity using magnetoencephalography. The next study will try to model the association (if any) between the functional and structural connectivity patterns found in the population. In parallel, the sample used in this study is currently enrolled in a longitudinal study to evaluate a possible clinical progression over time; therefore, some of the hypotheses presented could be better evaluated during the follow-up period. Second, machine learning techniques could also be evaluated to quantify nonlinear associations between subject characteristics and the presence of connectivity traits. Finally, the inclusion of additional neuroimaging techniques as tau-PET could help in the interpretation of the current results.

## Limitations

The main limitation related to the study design is the lack of A*β* biomarkers and tau biomarkers. Nevertheless, the population of this study are significantly younger than the average onset age of sporadic AD (Huff et al. 1987). Therefore, it is reasonable to suspect that the presence of Aβ would probably be in an oligomeric form, which is harder to detect (Yamin and Teplow 2017). The main limitation related to the methodological design is the instability or nondeterministic nature of the ICA. This limitation has been addressed enforcing the use of “robust” components as defined by (Amico et al. 2017).

## Supplementary Material

Ramirez_Torano_et_al_SM_R3_tgab051

## Data Availability

The data and the algorithms that support the findings of this study are available from the corresponding author, upon reasonable request.

## Notes

Thanks to all the experts and researchers that worked in this project. Thanks to all the researches in the CONNplexity lab that made this collaboration possible and fascinating. Also, thanks again to all the participants whose willingness to help is the basis of the project. *Conflict of Interest*: None declared. The funding sources had no role in the study design, data collection, data analyses, or data interpretation.
