# Supplementary material for: A Structural Connectivity Disruption One Decade before the Typical Age for Dementia: A Study in Healthy Subjects with Family History of Alzheimer’s Disease
Source: Cereb Cortex Commun. 2021 Aug 27;2(4):tgab051. doi: 10.1093/texcom/tgab051 (PMC8501268; doi:10.1093/texcom/tgab051)
Supplement: Ramirez_Torano_et_al_SM_R3_tgab051 [file ramirez_torano_et_al_sm_r3_tgab051.docx]

Supplementary Figure 1. Schaefer parcellation and matrix form.


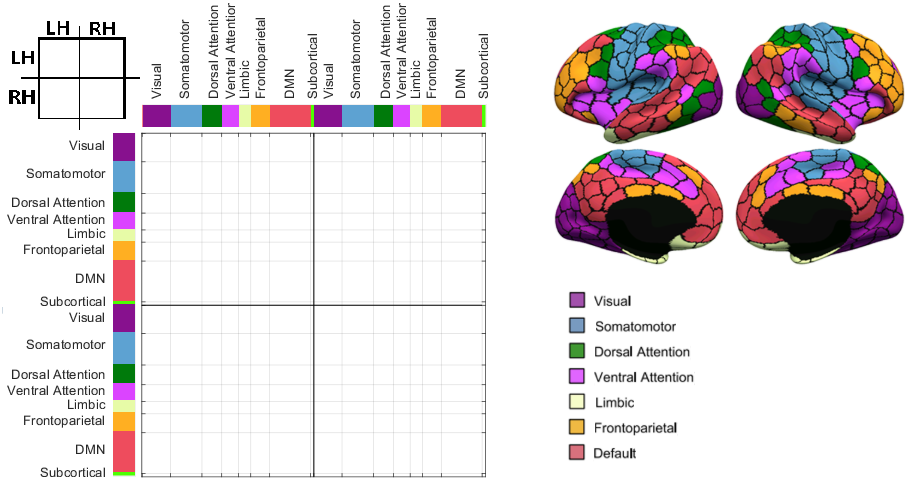


Supplementary Table 1. Multiple linear regression model statistics with the nine predictors.

|  | MLR model statistics | | | |
| --- | --- | --- | --- | --- |
|  | Standardized regression coefficients | $R^{2}$ gain | t-statistic | p-value |
| Age | -0.0926 ± 0.0361 | 0.0303 | -0.9468 | 0.3458 |
| Sex | 0.1512 ± 0.0517 | 0.0341 | 1.6530 | 0.1011 |
| Years of education | 0.0212 ± 0.0470 | 0.0016 | 0.2217 | 0.8250 |
| TPA | 0.1301 ± 0.0340 | 0.0189 | 1.5194 | 0.1315 |
| MoCA | 0.0129 ± 0.0555 | 0.0026 | 0.1401 | 0.8889 |
| Average cortical thickness | -0.0861 ± 0.0494 | 0.0046 | -0.9277 | 0.3556 |
| Hippocampus volume | 0.0917 ± 0.0514 | 0.0000 | 0.9201 | 0.3595 |
| *APOE* ε4 carriers | -0.0711 ± 0.0486 | 0.0040 | -0.8188 | 0.4146 |
| Family history of AD | 0.3423 ± 0.0372 | 0.1084 | 3.8713 | 0.0002 |

The values are presented as mean ± standard deviation

Supplementary Table 2. Demographic, neuropsychological, and neurophysiological data of each subject.

| Subject ID | Is relative | Age | Apoe carriage | Is male | Years of education | TPA | MoCA | MMSE | Average cortical thickness (mm) | Hippocampi volume (mm^3^) | Total Intracraneal Volume (mm^3^) | Temporal cortical thickness (mm)** |
| --- | --- | --- | --- | --- | --- | --- | --- | --- | --- | --- | --- | --- |
| fam_001 | 1 | 71 | 0 | 0 | 10 | 1.48 | 25 | 28 | 2.36 | 3,923.45 | 1,368,060 | 2.82 |
| fam_003 | 1 | 58 | 0 | 0 | 8 | 2.85 | 27 | 28 | 2.34 | 3,790.65 | 1,319,020 | 2.83 |
| fam_004 | 1 | 70 | 0 | 1 | 11 | 0.59 | 30 | 30 | 2.39 | 3,504.50 | 1,298,220 | 2.81 |
| fam_005 | 1 | 51 | 0 | 0 | 12 | 1.83 | 24 | 29 | 2.38 | 3,900.55 | 1,356,610 | 2.69 |
| fam_006 | 1 | 65 | 1 | 0 | 20 | 0.41 | 28 | 30 | 2.32 | 3,778.50 | 1,433,990 | 2.80 |
| fam_007 | 1 | 57 | 1 | 0 | 14 | 0.90 | 30 | 27 | 2.48 | 3,904.85 | 1,323,360 | 2.92 |
| fam_008 | 1 | 58 | 1 | 0 | 16 | 1.48 | 25 | 30 | 2.46 | 4,293.95 | 1,424,860 | 2.92 |
| fam_009 | 1 | 64 | 1 | 0 | 16 | 4.43 | 23 | 30 | 2.47 | 3,748.20 | 1,320,450 | 2.96 |
| fam_010 | 1 | 62 | 0 | 0 | 19 | 1.08 | 30 | 29 | 2.40 | 2,915.40 | 1,250,180 | 2.84 |
| fam_011 | 1 | 54 | 0 | 0 | 19 | 2.96 | 30 | 29 | 2.39 | 3,695.35 | 1,297,160 | 2.88 |
| fam_012 | 1 | 53 | 0 | 1 | 14 | 2.11 | 24 | 29 | 2.25 | 4,124.40 | 1,580,360 | 2.66 |
| fam_013 | 1 | 44 | 1 | 1 | 18 | 1.48 | 28 | 29 | 2.51 | 4,584.70 | 1,620,110 | 3.01 |
| fam_014 | 1 | 70 | 0 | 0 | 16 | 0.36 | 30 | 30 | 2.17 | 3,343.10 | 1,404,790 | 2.71 |
| fam_015 | 1 | 62 | 0 | 0 | 12 | 1.48 | 25 | 26 | 2.47 | 3,656.70 | 1,309,640 | 3.02 |
| fam_016 | 1 | 43 | 1 | 1 | 20 | 0.24 | 29 | 30 | 2.38 | 4,160.95 | 1,543,070 | 2.80 |
| fam_017 | 1 | 57 | 1 | 0 | 21 | 0.37 | 28 | 30 | 2.34 | 4,083.65 | 1,462,740 | 2.83 |
| fam_019 | 1 | 66 | 0 | 0 | 13 | 0.89 | 28 | 30 | 2.30 | 3,548.20 | 1,245,310 | 2.78 |
| fam_020 | 1 | 55 | 0 | 0 | 14 | 3.05 | 29 | 28 | 2.33 | 3,198.40 | 1,035,420 | 2.83 |
| fam_021 | 1 | 41 | 1 | 0 | 17 | 1.48 | 29 | 28 | 2.44 | 4,072.45 | 1,381,790 | 3.10 |
| fam_023 | 1 | 53 | 1 | 0 | 16 | 1.54 | 29 | 30 | 2.29 | 3,111.80 | 1,486,850 | 2.79 |
| fam_024 | 1 | 56 | 1 | 0 | 15 | 0.11 | 27 | 29 | 2.51 | 3,243.00 | 1,178,870 | 2.87 |
| fam_025 | 1 | 66 | 0 | 0 | 15 | 1.82 | 23 | 29 | 2.36 | 3,362.15 | 1,367,920 | 2.96 |
| fam_028 | 1 | 66 | 0 | 0 | 13 | 2.87 | 25 | 29 | 2.35 | 3,960.15 | 1,289,990 | 2.85 |
| fam_029 | 1 | 54 | 1 | 0 | 17 | 0.38 | 27 | 30 | 2.32 | 3,627.45 | 1,234,370 | 2.90 |
| fam_030 | 0 | 68 | 0 | 0 | 17 | 0.20 | 29 | 30 | 2.29 | 3,514.05 | 1,308,880 | 2.73 |
| fam_033 | 1 | 61 | 1 | 0 | 20 | 0.10 | N/A* | N/A* | 2.38 | 3,277.55 | 1,253,030 | 2.89 |
| fam_034 | 1 | 58 | 0 | 1 | 17 | 6.42 | 29 | 30 | 2.43 | 4,059.80 | 1,612,830 | 2.99 |
| fam_035 | 1 | 52 | 1 | 0 | 20 | 0.85 | 27 | 28 | 2.30 | 4,092.95 | 1,407,070 | 2.84 |
| fam_038 | 1 | 45 | 0 | 0 | 17 | 0.26 | 27 | 29 | 2.33 | 3,569.10 | 1,317,500 | 2.75 |
| fam_039 | 1 | 50 | 1 | 0 | 12 | 1.48 | 24 | 30 | 2.38 | 3,770.50 | 1,339,390 | 2.80 |
| fam_040 | 1 | 70 | 0 | 0 | 5 | 0.10 | 18 | 26 | 2.44 | 3,292.50 | 1,219,250 | 2.90 |
| fam_042 | 1 | 57 | 0 | 0 | 19 | 0.11 | 28 | 28 | 2.33 | 3,589.95 | 1,522,570 | 2.75 |
| fam_043 | 1 | 55 | 1 | 0 | 18 | 0.74 | 25 | 30 | 2.40 | 4,159.10 | 1,395,680 | 2.82 |
| fam_045 | 1 | 62 | 0 | 1 | 16 | 0.77 | 27 | 30 | 2.39 | 4,137.60 | 1,429,790 | 2.94 |
| fam_046 | 0 | 56 | 0 | 1 | 11 | 1.48 | 21 | 29 | 2.37 | 5,051.50 | 1,327,090 | 2.89 |
| fam_047 | 1 | 52 | 0 | 1 | 18 | 3.71 | 26 | 30 | 2.39 | 3,948.55 | 1,568,270 | 2.90 |
| fam_048 | 0 | 79 | 0 | 0 | 19 | 0.60 | N/A* | 28 | 2.15 | 2,840.20 | 1,110,930 | 2.41 |
| fam_049 | 1 | 60 | 0 | 0 | 14 | 0.56 | N/A* | 29 | 2.45 | 3,958.70 | 1,252,630 | 2.88 |
| fam_050 | 1 | 62 | 1 | 1 | 17 | 1.77 | N/A* | 30 | 2.38 | 3,945.70 | 1,501,630 | 2.85 |
| fam_051 | 1 | 64 | 1 | 1 | 21 | 2.55 | 25 | 28 | 2.31 | 3,757.00 | 1,545,140 | 2.84 |
| fam_052 | 1 | 54 | 0 | 0 | 20 | 0.28 | 30 | 30 | 2.43 | 4,455.95 | 1,447,800 | 2.84 |
| fam_053 | 0 | 77 | 0 | 1 | 20 | 0.60 | 26 | 30 | 2.33 | 3,998.25 | 1,661,320 | 2.73 |
| fam_054 | 0 | 81 | 0 | 0 | 16 | 3.71 | 27 | 29 | 2.27 | 3,450.25 | 1,414,670 | 2.69 |
| fam_055 | 1 | 64 | 0 | 1 | 21 | 0.72 | 28 | 29 | 2.30 | 3,532.80 | 1,461,700 | 2.74 |
| fam_056 | 0 | 68 | 0 | 0 | 14 | 1.48 | N/A* | 30 | 2.29 | 3,158.65 | 1,278,110 | 2.86 |
| fam_058 | 0 | 69 | 1 | 0 | 14 | 1.48 | N/A* | 30 | 2.31 | 3,142.80 | 1,411,570 | 2.71 |
| fam_059 | 1 | 55 | 1 | 1 | 21 | 1.48 | 26 | 30 | 2.33 | 3,940.70 | 1,527,820 | 2.85 |
| fam_060 | 1 | 36 | 1 | 0 | 13 | 1.48 | 26 | 29 | 2.41 | 3,764.25 | 1,407,980 | 2.82 |
| fam_062 | 0 | 74 | 0 | 0 | 16 | 0.12 | N/A* | 30 | 2.40 | 3,820.65 | 1,285,930 | 2.99 |
| fam_066 | 1 | 68 | 1 | 0 | 16 | 0.38 | 25 | 28 | 2.37 | 3,176.05 | 1,103,760 | 2.72 |
| fam_067 | 1 | 70 | 1 | 0 | 10 | 1.48 | 19 | 26 | 2.39 | 3,334.10 | 1,341,830 | 2.82 |
| fam_068 | 1 | 62 | 0 | 0 | 10 | 2.28 | 20 | 29 | 2.31 | 3,525.65 | 1,304,980 | 2.72 |
| fam_069 | 0 | 52 | 0 | 0 | 18 | 0.57 | 30 | 30 | 2.44 | 4,109.05 | 1,428,690 | 3.00 |
| fam_071 | 1 | 55 | 0 | 0 | 16 | 0.12 | 28 | 30 | 2.62 | 3,802.75 | 1,387,570 | 3.02 |
| fam_072 | 1 | 59 | 0 | 0 | 22 | 5.29 | 29 | 29 | 2.40 | 3,484.15 | 1,231,930 | 2.77 |
| fam_074 | 1 | 58 | 0 | 0 | 11 | 0.24 | 23 | 29 | 2.29 | 3,800.25 | 1,278,110 | 2.86 |
| fam_075 | 0 | 60 | 1 | 1 | 14 | 0.25 | 27 | 30 | 2.41 | 3,691.55 | 1,313,960 | 2.92 |
| fam_076 | 1 | 51 | 1 | 1 | 10 | 1.48 | 30 | 30 | 2.32 | 3,842.05 | 1,576,630 | 2.81 |
| fam_077 | 1 | 61 | 0 | 0 | 22 | 0.77 | 22 | 30 | 2.42 | 3,931.35 | 1,246,730 | 2.85 |
| fam_078 | 0 | 65 | 1 | 0 | 19 | 0.68 | N/A* | 30 | 2.52 | 3,198.35 | 1,201,800 | 3.00 |
| fam_080 | 1 | 60 | 0 | 0 | 20 | 0.35 | 30 | 30 | 2.41 | 3,875.95 | 1,466,520 | 2.93 |
| fam_081 | 1 | 68 | 0 | 0 | 10 | 1.48 | 27 | 27 | 2.32 | 3,749.45 | 1,325,800 | 2.80 |
| fam_082 | 1 | 61 | 1 | 0 | 18 | 1.48 | 21 | 29 | 2.25 | 3,463.70 | 1,193,810 | 2.69 |
| fam_083 | 1 | 62 | 0 | 1 | 11 | 1.48 | 22 | 29 | 2.28 | 3,614.25 | 1,407,850 | 2.68 |
| fam_084 | 1 | 61 | 0 | 0 | 16 | 1.48 | 28 | 29 | 2.37 | 3,569.45 | 1,273,450 | 2.84 |
| fam_085 | 1 | 56 | 1 | 0 | 16 | 1.48 | 30 | 30 | 2.47 | 3,490.00 | 1,314,050 | 2.94 |
| fam_086 | 1 | 60 | 1 | 0 | 18 | 1.64 | 26 | 29 | 2.43 | 4,018.95 | 1,462,380 | 2.78 |
| fam_087 | 1 | 55 | 1 | 0 | 15 | 0.20 | 30 | 30 | 2.37 | 4,134.50 | 1,401,360 | 2.78 |
| fam_089 | 1 | 55 | 1 | 0 | 16 | 1.22 | 25 | 29 | 2.34 | 4,089.00 | 1,392,440 | 2.87 |
| fam_090 | 1 | 59 | 1 | 1 | 14 | 1.48 | 26 | 29 | 2.23 | 3,777.05 | 1,498,900 | 2.64 |
| fam_092 | 1 | 50 | 0 | 0 | 14 | 2.54 | 24 | 28 | 2.53 | 4,432.80 | 1,392,110 | 3.10 |
| fam_093 | 1 | 49 | 0 | 1 | 16 | 2.10 | 27 | 30 | 2.41 | 4,585.85 | 1,375,110 | 2.98 |
| fam_094 | 0 | 66 | 0 | 0 | 21 | 1.48 | 30 | 30 | 2.36 | 3,939.60 | 1,319,150 | 2.73 |
| fam_095 | 0 | 77 | 0 | 0 | 12 | 0.26 | 26 | 29 | 2.38 | 3,748.55 | 1,389,840 | 2.87 |
| fam_096 | 0 | 62 | 1 | 0 | 11 | 1.76 | 23 | 28 | 2.35 | 3,348.40 | 1,222,370 | 2.92 |
| fam_097 | 1 | 70 | 1 | 0 | 11 | 0.27 | 23 | 28 | 2.39 | 3,335.20 | 1,252,690 | 2.79 |
| fam_098 | 0 | 51 | 0 | 0 | 17 | 2.27 | 24 | 30 | 2.49 | 3,845.45 | 1,376,030 | 2.85 |
| fam_099 | 1 | 52 | 0 | 1 | 18 | 0.93 | 25 | 30 | 2.41 | 3,797.91 | 1,766,110 | 2.93 |
| fam_101 | 1 | 60 | 0 | 0 | 18 | 2.95 | 23 | 30 | 2.31 | 4,804.70 | 1,602,860 | 2.82 |
| fam_102 | 0 | 69 | 0 | 1 | 21 | 4.84 | 29 | 29 | 2.33 | 3,538.05 | 1,404,410 | 2.79 |
| fam_103 | 0 | 82 | 1 | 0 | 9 | 1.48 | 21 | 29 | 2.15 | 2,703.55 | 1,397,260 | 2.46 |
| fam_104 | 1 | 51 | 0 | 0 | 11 | 3.84 | 30 | 30 | 2.49 | 3,555.15 | 1,254,300 | 2.85 |
| fam_105 | 0 | 53 | 0 | 1 | 12 | 0.65 | 26 | 28 | 2.37 | 4,415.45 | 1,501,000 | 2.81 |
| fam_106 | 0 | 62 | 0 | 1 | 19 | 0.80 | 26 | 30 | 2.36 | 4,068.30 | 1,398,080 | 2.93 |
| fam_107 | 1 | 59 | 0 | 0 | 20 | 2.21 | 30 | 29 | 2.36 | 3,625.15 | 1,416,180 | 2.90 |
| fam_108 | 1 | 52 | 0 | 1 | 20 | 3.39 | 26 | 29 | 2.40 | 4,820.95 | 1,778,930 | 2.93 |
| fam_109 | 1 | 56 | 0 | 1 | 14 | 0.68 | 26 | 29 | 2.26 | 4,110.50 | 1,650,170 | 2.77 |
| fam_112 | 1 | 63 | 1 | 0 | 21 | 1.39 | 28 | 29 | 2.51 | 3,621.05 | 1,328,180 | 3.02 |
| fam_113 | 0 | 56 | 0 | 0 | 17 | 1.48 | 25 | 28 | 2.31 | 3,331.70 | 1,355,890 | 2.76 |
| fam_115 | 1 | 67 | 0 | 1 | 14 | 0.56 | 23 | 29 | 2.32 | 4,096.50 | 1,522,960 | 2.74 |
| fam_119 | 1 | 57 | 1 | 1 | 9 | 1.48 | 29 | 29 | 2.25 | 3,309.95 | 1,609,040 | 2.70 |
| fam_120 | 0 | 55 | 0 | 0 | 7 | 1.48 | 26 | 29 | 2.46 | 3,752.15 | 1,512,460 | 3.08 |
| fam_121 | 0 | 62 | 1 | 0 | 18 | 3.36 | 28 | 24 | 2.35 | 3,733.60 | 1,274,160 | 2.86 |
| fam_123 | 0 | 53 | 0 | 0 | 17 | 1.77 | 28 | 30 | 2.35 | 3,500.40 | 1,287,060 | 2.76 |
| fam_124 | 1 | 62 | 0 | 1 | 19 | 0.88 | 26 | 30 | 2.38 | 3,472.15 | 1,252,780 | 2.89 |
| fam_125 | 1 | 57 | 0 | 1 | 13 | 0.18 | 23 | 29 | 2.33 | 3,971.45 | 1,434,470 | 2.71 |
| fam_126 | 0 | 61 | 0 | 0 | 18 | 2.83 | 24 | 29 | 2.28 | 3,998.25 | 1,450,310 | 2.79 |
| fam_127 | 0 | 54 | 0 | 1 | 9 | 2.21 | 24 | 28 | 2.42 | 3,454.05 | 1,359,060 | 2.97 |
| fam_128 | 1 | 60 | 0 | 0 | 17 | 0.48 | 26 | 30 | 2.41 | 3,731.65 | 1,304,270 | 2.92 |
| fam_129 | 0 | 50 | 0 | 1 | 18 | 0.20 | 26 | 30 | 2.27 | 3,795.45 | 1,326,850 | 2.81 |
| fam_131 | 1 | 62 | 0 | 0 | 15 | 5.51 | 21 | 22 | 2.34 | 3,797.91 | 1,369,870 | 2.73 |
| fam_132 | 1 | 51 | 1 | 0 | 14 | 1.52 | 29 | 30 | 2.34 | 3,880.25 | 1,354,980 | 2.71 |
| fam_133 | 1 | 60 | 0 | 1 | 21 | 2.23 | 25 | 29 | 2.29 | 4,320.00 | 1,652,740 | 2.90 |
| fam_134 | 1 | 52 | 1 | 0 | 16 | 0.57 | 27 | 29 | 2.38 | 4,175.95 | 1,465,350 | 2.90 |
| fam_135 | 0 | 77 | 0 | 0 | 20 | 1.48 | N/A* | 30 | 2.40 | 4,132.45 | 1,330,600 | 2.91 |
| fam_136 | 0 | 74 | 0 | 1 | 20 | 2.22 | N/A* | 28 | 2.25 | 3,825.90 | 1,545,670 | 2.72 |
| fam_137 | 0 | 60 | 0 | 0 | 19 | 1.80 | 27 | 29 | 2.51 | 4,130.05 | 1,369,710 | 3.07 |
| fam_138 | 0 | 70 | 0 | 0 | 17 | 1.48 | N/A* | 30 | 2.28 | 3,018.30 | 1,351,580 | 2.49 |
| fam_139 | 0 | 68 | 1 | 0 | 13 | 1.48 | N/A* | 30 | 2.38 | 2,908.50 | 1,301,260 | 2.83 |
| fam_141 | 1 | 56 | 0 | 0 | 13 | 2.14 | 29 | 29 | 2.42 | 3,976.75 | 1,655,330 | 2.83 |
| fam_142 | 1 | 51 | 0 | 0 | 18 | 4.60 | 30 | 30 | 2.39 | 3,852.55 | 1,421,860 | 2.86 |
| fam_143 | 1 | 49 | 0 | 0 | 18 | 1.22 | 26 | 29 | 2.35 | 3,924.70 | 1,474,680 | 2.80 |
| fam_144 | 0 | 68 | 1 | 1 | 13 | 1.36 | 27 | 28 | 2.38 | 3,976.60 | 1,321,670 | 2.87 |
| fam_145 | 0 | 62 | 0 | 1 | 16 | 1.48 | 27 | 29 | 2.30 | 3,274.35 | 1,191,860 | 2.77 |
| fam_146 | 1 | 58 | 1 | 0 | 17 | 0.62 | 26 | 29 | 2.40 | 4,107.80 | 1,459,890 | 2.77 |
| fam_147 | 0 | 59 | 1 | 0 | 21 | 3.80 | 26 | 29 | 2.39 | 3,742.95 | 1,304,320 | 2.84 |
| fam_148 | 1 | 53 | 0 | 1 | 17 | 0.50 | 26 | 29 | 2.36 | 4,130.80 | 1,462,400 | 2.82 |
| fam_149 | 0 | 54 | 0 | 1 | 17 | 0.55 | 29 | 29 | 2.37 | 3,897.90 | 1,628,250 | 2.93 |
| fam_151 | 1 | 58 | 0 | 0 | 8 | 0.48 | 22 | 26 | 2.41 | 4,064.00 | 1,263,280 | 2.91 |
| fam_152 | 1 | 50 | 1 | 0 | 17 | 0.59 | 25 | 29 | 2.39 | 3,406.65 | 1,502,650 | 2.82 |
| fam_153 | 1 | 56 | 1 | 0 | 15 | 0.91 | 27 | 30 | 2.37 | 3,779.20 | 1,370,740 | 2.87 |
| fam_154 | 1 | 53 | 0 | 1 | 11 | 1.48 | 27 | 29 | 2.25 | 4,367.50 | 1,541,780 | 2.93 |
| fam_155 | 1 | 50 | 1 | 1 | 11 | 0.61 | 25 | 30 | 2.27 | 4,079.10 | 1,522,110 | 2.80 |
| fam_157 | 0 | 76 | 0 | 1 | 20 | 0.10 | 24 | 28 | 2.28 | 2,774.25 | 1,201,610 | 2.69 |
| fam_158 | 0 | 48 | 0 | 0 | 14 | 0.58 | 25 | 28 | 2.35 | 3,907.45 | 1,307,700 | 2.87 |
| fam_159 | 1 | 62 | 0 | 0 | 15 | 1.71 | 24 | 30 | 2.37 | 3,936.35 | 1,351,690 | 2.84 |
| fam_160 | 0 | 60 | 0 | 0 | 17 | 2.31 | 25 | 28 | 2.34 | 3,769.30 | 1,185,040 | 2.80 |
| fam_161 | 0 | 52 | 0 | 0 | 19 | 0.75 | 25 | 30 | 2.47 | 3,695.25 | 1,482,650 | 2.88 |
| fam_162 | 1 | 56 | 1 | 0 | 16 | 0.62 | 25 | 28 | 2.49 | 3,348.45 | 1,278,550 | 2.99 |
| fam_163 | 1 | 50 | 0 | 0 | 17 | 1.86 | 23 | 28 | 2.21 | 3,735.00 | 1,614,690 | 2.61 |
| fam_164 | 0 | 51 | 0 | 0 | 19 | 0.10 | 30 | 29 | 2.35 | 3,629.70 | 1,438,640 | 2.84 |
| fam_165 | 1 | 63 | 0 | 0 | 17 | 3.41 | 27 | 29 | 2.29 | 3,370.45 | 1,224,400 | 2.87 |
| fam_166 | 1 | 56 | 1 | 1 | 19 | 3.25 | 27 | 28 | 2.47 | 4,824.80 | 1,673,740 | 3.07 |
| fam_168 | 1 | 55 | 0 | 0 | 11 | 1.48 | 23 | 27 | 2.30 | 3,997.55 | 1,454,840 | 2.75 |
| fam_169 | 1 | 67 | 1 | 0 | 24 | 1.48 | 27 | 30 | 2.42 | 4,035.40 | 1,329,900 | 2.95 |
| fam_170 | 1 | 65 | 0 | 1 | 18 | 0.84 | 22 | 26 | 2.32 | 3,476.50 | 1,465,100 | 2.75 |
| fam_172 | 1 | 58 | 1 | 0 | 15 | 0.31 | 26 | 30 | 2.43 | 4,048.55 | 1,261,010 | 2.95 |
| fam_175 | 1 | 49 | 0 | 0 | 20 | 0.21 | 29 | 30 | 2.41 | 3,440.10 | 1,041,480 | 2.84 |
| fam_176 | 1 | 55 | 0 | 0 | 16 | 2.81 | 24 | 26 | 2.35 | 3,485.80 | 1,424,440 | 2.83 |
| fam_177 | 0 | 65 | 0 | 1 | 16 | 3.37 | N/A* | 30 | 2.27 | 3,795.35 | 1,715,740 | 2.74 |
| fam_178 | 1 | 62 | 0 | 0 | 11 | 0.22 | 25 | 29 | 2.43 | 3,523.35 | 1,269,390 | 2.75 |
| fam_179 | 0 | 53 | 1 | 1 | 19 | 0.70 | 28 | 30 | 2.34 | 5,029.45 | 2,006,110 | 2.88 |
| fam_180 | 1 | 64 | 1 | 0 | 9 | 2.24 | 21 | 25 | 2.36 | 3,224.30 | 1,354,900 | 2.74 |
| fam_181 | 1 | 52 | 0 | 1 | 17 | 1.18 | 30 | 30 | 2.39 | 3,993.95 | 1,563,110 | 2.94 |
| fam_182 | 0 | 69 | 0 | 1 | 21 | 0.13 | 25 | 28 | 2.19 | 3,763.75 | 1,839,880 | 2.63 |
| fam_183 | 1 | 71 | 0 | 0 | 7 | 0.84 | 20 | 29 | 2.41 | 3,925.60 | 1,325,770 | 2.98 |
| fam_184 | 0 | 53 | 0 | 0 | 20 | 1.50 | N/A* | 30 | 2.50 | 3,969.60 | 1,317,940 | 3.06 |
| fam_185 | 0 | 55 | 0 | 1 | 20 | 2.07 | 28 | 28 | 2.36 | 4,046.55 | 1,503,230 | 2.84 |
| fam_186 | 1 | 55 | 0 | 0 | 20 | 1.15 | 29 | 29 | 2.31 | 3,666.95 | 1,284,050 | 2.75 |
| fam_187 | 1 | 53 | 1 | 1 | 13 | 3.70 | 26 | 29 | 2.30 | 4,300.40 | 1,426,530 | 2.74 |
| fam_188 | 0 | 56 | 0 | 1 | 19 | 1.22 | 25 | 29 | 2.39 | 4,003.45 | 1,271,260 | 2.85 |
| fam_189 | 1 | 57 | 0 | 0 | 6 | 1.68 | 22 | 28 | 2.43 | 3,562.65 | 1,325,350 | 2.78 |
| fam_190 | 0 | 70 | 0 | 1 | 21 | 1.85 | 28 | 30 | 2.37 | 3,836.70 | 1,464,550 | 2.92 |
| fam_191 | 1 | 57 | 0 | 1 | 20 | 1.48 | 29 | 30 | 2.31 | 4,309.40 | 1,651,170 | 2.81 |
| fam_193 | 0 | 53 | 1 | 1 | 19 | 1.15 | 24 | 30 | 2.41 | 4,139.30 | 1,621,210 | 2.93 |
| fam_194 | 1 | 67 | 0 | 1 | 15 | 1.48 | 27 | 29 | 2.49 | 4,096.65 | 1,554,760 | 2.89 |
| fam_195 | 0 | 59 | 0 | 0 | 12 | 1.48 | 24 | 30 | 2.29 | 3,703.75 | 1,275,450 | 2.81 |
| fam_196 | 1 | 61 | 0 | 1 | 11 | 1.48 | 23 | 27 | 2.35 | 3,728.00 | 1,445,840 | 2.84 |
| fam_197 | 0 | 59 | 0 | 0 | 12 | 2.87 | 26 | 28 | 2.28 | 4,058.05 | 1,462,760 | 2.80 |
| fam_198 | 1 | 63 | 0 | 0 | 15 | 0.48 | 20 | 28 | 2.23 | 4,393.65 | 1,499,070 | 2.79 |
| fam_199 | 1 | 72 | 0 | 1 | 16 | 1.58 | 27 | 29 | 2.44 | 4,259.60 | 1,417,220 | 3.02 |
| fam_200 | 0 | 68 | 0 | 1 | 18 | 1.48 | 28 | 29 | 2.42 | 4,278.80 | 1,495,300 | 2.95 |
| fam_201 | 0 | 63 | 0 | 1 | 23 | 1.04 | 21 | 29 | 2.42 | 3,826.70 | 1,421,050 | 2.95 |
| fam_202 | 0 | 51 | 0 | 0 | 21 | 1.24 | 25 | 29 | 2.45 | 3,806.90 | 1,140,050 | 2.87 |
| fam_204 | 1 | 51 | 0 | 1 | 19 | 0.87 | 29 | 30 | 2.34 | 3,998.55 | 1,581,660 | 2.87 |
| fam_205 | 0 | 77 | 0 | 0 | 8 | 0.46 | 26 | 30 | 2.35 | 3,799.75 | 1,318,800 | 2.87 |
| fam_206 | 0 | 55 | 0 | 0 | 22 | 3.08 | N/A* | N/A* | 2.44 | 4,123.45 | 1,583,750 | 2.99 |
| fam_207 | 0 | 80 | 0 | 1 | 21 | 5.59 | 21 | 29 | 2.35 | 4,145.30 | 1,738,230 | 3.03 |
| fam_209 | 1 | 55 | 0 | 1 | 16 | 0.10 | 25 | 30 | 2.37 | 3,571.85 | 1,533,360 | 2.99 |
| fam_210 | 1 | 68 | 0 | 0 | 14 | 0.24 | 25 | 29 | 2.41 | 4,100.90 | 1,424,520 | 2.82 |
| fam_211 | 1 | 53 | 0 | 1 | 18 | 1.48 | 28 | 28 | 2.33 | 4,276.80 | 1,491,830 | 2.91 |
| fam_212 | 1 | 58 | 0 | 0 | 16 | 1.07 | 26 | 29 | 2.34 | 3,621.05 | 1,275,320 | 2.86 |
| fam_213 | 0 | 56 | 0 | 0 | 22 | 2.04 | 30 | 28 | 2.34 | 3,981.75 | 1,516,590 | 2.67 |
| fam_214 | 1 | 59 | 0 | 0 | 19 | 1.28 | 29 | 30 | 2.37 | 3,357.70 | 1,287,070 | 2.87 |
| fam_215 | 0 | 58 | 1 | 0 | 18 | 1.48 | 28 | 28 | 2.28 | 3,572.00 | 1,326,900 | 2.76 |
| fam_217 | 1 | 50 | 1 | 1 | 20 | 1.20 | 28 | 30 | 2.44 | 4,123.45 | 1,583,750 | 2.99 |
| fam_218 | 0 | 53 | 0 | 1 | 19 | 2.59 | 30 | 29 | 2.33 | 3,729.75 | 1,420,340 | 2.71 |
| fam_220 | 1 | 53 | 1 | 1 | 14 | 1.48 | 27 | 28 | 2.42 | 3,797.91 | 1,258,770 | 3.02 |
| fam_221 | 1 | 70 | 0 | 1 | 9 | 1.15 | 27 | 29 | 2.37 | 3,018.55 | 1,575,160 | 2.80 |
| fam_222 | 0 | 68 | 1 | 0 | 12 | 0.81 | 16 | 23 | 2.35 | 3,456.80 | 1,348,880 | 2.88 |
| fam_228 | 1 | 51 | 0 | 0 | 18 | 0.48 | 30 | 30 | 2.47 | 3,960.35 | 1,272,140 | 3.01 |
| fam_229 | 1 | 54 | 1 | 1 | 16 | 0.27 | 27 | 30 | 2.31 | 3,550.35 | 1,409,970 | 2.91 |
| fam_230 | 0 | 64 | 0 | 1 | 20 | 0.68 | 29 | 30 | 2.31 | 3,934.90 | 1,469,870 | 2.73 |
| fam_231 | 1 | 70 | 0 | 1 | 8 | 1.48 | 23 | 27 | 2.29 | 3,322.75 | 1,410,810 | 2.63 |

* Some neuropsychological tests are not available for some subjects. Based on the complete neuropsychological assessment of these subjects performed by Silvia Marcos de Pedro, these subjects do not meet the diagnosis criteria for AD, MCI, or preclinical stages of AD.

** Mean cortical thickness of the areas entorhinal, fusiform, inferior temporal and middle temporal as estimated in (Jack *et al.*, 2017).
